# Effect of Orthodontic Tooth Movement on Pulp Vitality in Previously Traumatized Teeth: A Prospective Cohort Study

**DOI:** 10.7759/cureus.105938

**Published:** 2026-03-26

**Authors:** Sarmishtha Boruah, Ikroop Gill, Navleen Dandiwal, Manoj K Sharma, Ishu Agarwal, Arti Devi, Seema Gupta

**Affiliations:** 1 Department of Conservative Dentistry and Endodontics, Government Dental College, Dibrugarh, IND; 2 Department of Conservative Dentistry and Endodontics, All India Institute of Medical Sciences, Bathinda, IND; 3 Department of Dentistry, All India Institute of Medical Sciences, Bathinda, IND; 4 Department of Orthodontics, Kothiwal Dental College and Research Centre, Moradabad, IND; 5 Department of Orthodontics, Maharishi Markandeshwar College of Dental Sciences and Research (MMCDSR), Ambala, IND

**Keywords:** calcification, orthodontic, pulp vitality, tooth movement, trauma

## Abstract

Introduction: Dental trauma commonly affects the anterior teeth of young individuals, often leading to pulpal compromise through neurovascular damage. Orthodontic tooth movement (OTM) may further stress these teeth, thereby increasing the risk of loss of vitality. This prospective cohort study aimed to assess the impact of OTM on pulp vitality in previously traumatized permanent anterior teeth compared to non-traumatized contralateral controls.

Materials and methods: Sixty patients (mean age: 16.5 ± 3.2 years) participated, each providing one traumatized anterior tooth (traumatized group) and one non-traumatized contralateral tooth (control group) treated with fixed orthodontics. The trauma types included enamel-dentin fractures, subluxation, and enamel-dentin-pulp fractures, with a mean time since injury of 3.8 ± 2.1 years. Pulp vitality was evaluated using cold and electric pulp testing at baseline and at 3, 6, and 12 months after OTM initiation. Data were analyzed using McNemar’s test, Kaplan-Meier survival analysis with log-rank test, and relative risk estimation (p < 0.05).

Results: Baseline vitality was positive in 58 (96.7%) traumatized teeth and 60 (100%) control teeth (p = 0.154). Vitality in the traumatized group declined to 54 (90.0%) at three months (p = 0.047), 50 (83.3%) at six months (p = 0.015), and 48 (80.0%) at 12 months (p = 0.013), while the controls showed minimal changes. Kaplan-Meier survival analysis revealed a mean vitality survival of 10.8 months in traumatized teeth versus 11.8 months in controls (log-rank p = 0.016). Pulp necrosis occurred in eight (13.3%) traumatized teeth versus one (1.7%) control tooth (p = 0.035). Calcific metamorphosis was observed in four (6.7%) and in none of the controls (p = 0.126). The external root resorption rates were similar.

Conclusion: OTM significantly increased the risk of pulp vitality loss and pulpal complications in previously traumatized teeth. These findings highlight the need for cautious interdisciplinary management, including the use of light orthodontic forces (≤25-50 g), avoidance of heavy intrusive movements, regular pulp sensibility monitoring at baseline and at three-month intervals, and thorough patient counseling regarding the risk of pulp necrosis and possible endodontic intervention. Multicenter studies using objective pulpal blood flow assessment methods are recommended to strengthen evidence-based protocols.

## Introduction

Dental trauma is a prevalent condition, particularly among children and adolescents, and often affects the anterior teeth owing to their prominent position in the dental arch. Common injuries include luxation (intrusive, extrusive, and lateral), avulsion, crown fractures, and root fractures, which can compromise both the periodontal ligament and the dental pulp [1]. These traumas may lead to immediate or delayed complications such as pulp necrosis, pulp canal obliteration, root resorption, and loss of tooth vitality [2]. In many cases, patients with a history of dental trauma subsequently require orthodontic treatment to correct malocclusions, close spaces, or improve aesthetics and function following trauma-related tooth displacement or loss [3].

Orthodontic tooth movement (OTM) relies on controlled mechanical forces applied to the teeth, inducing remodeling of the periodontal ligament, alveolar bone, and surrounding tissues [4]. Although OTM is generally considered safe for healthy vital teeth, it can temporarily reduce pulpal blood flow, alter pulp sensibility, and induce inflammatory responses in the pulp-dentine complex. In non-traumatized teeth, these changes are usually reversible, with pulp vitality being preserved under light and physiological forces [5]. However, teeth with a history of trauma present a unique challenge at the orthodontic-endodontic interface. Trauma often damages the neurovascular supply to the pulp, leading to partial or complete pulp obliteration, compromised vascularity, or subclinical pulp compromise [6]. When orthodontic forces, particularly intrusive, extrusive, or heavy forces, are applied to such teeth, they may further impair pulpal circulation, increasing the susceptibility to complications, such as pulp necrosis or delayed vitality loss [6,7].

Previous studies have consistently highlighted an elevated risk of pulp necrosis in traumatized teeth undergoing OTM compared to non-traumatized controls [6-8]. Factors such as the severity of the initial trauma (especially those involving periodontal tissues such as luxation or intrusion), presence of total pulp obliteration, type and magnitude of orthodontic forces, maturity of the tooth apex, and interval between trauma and orthodontic initiation appear to influence outcomes [8]. For instance, severe periodontal injuries and shorter post-trauma waiting periods have been associated with higher rates of non-vitality. Despite these observations, evidence remains moderate at best, with calls for more robust clinical data to guide interdisciplinary protocols, including optimal force levels, monitoring strategies, and waiting periods [9]. However, there is a paucity of studies employing a paired split-mouth cohort design with structured longitudinal follow-up, which allows direct intra-individual comparison and minimizes confounding; the present study was designed to address this gap.

The present study aimed to evaluate the effect of OTM on pulp vitality in previously traumatized teeth. It was hypothesized that traumatized teeth would demonstrate a higher risk of pulp vitality loss compared to non-traumatized contralateral controls. The specific objectives were to (1) compare pulp vitality outcomes between traumatized and control teeth to assess clinical risk, (2) evaluate the influence of trauma-related and treatment-related factors on pulpal response to guide clinical decision-making, and (3) assess implications for interdisciplinary management to improve patient outcomes.

## Materials and methods

This study was designed as a prospective cohort study and was conducted at the Kothiwal Dental College and Research Centre, Moradabad, Uttar Pradesh, India, from May 2024 to September 2025. Ethical approval was obtained from the Institutional Ethical Review Board (KDCRC/IERB/4/2024/SS12) of Kothiwal Dental College and Research Centre before data collection. Strict confidentiality protocols were followed throughout the study. All data were de-identified, stored securely, and used solely for research purposes in accordance with institutional guidelines and the principles of the Declaration of Helsinki.

The eligibility criteria were clearly defined to ensure the inclusion of appropriate cases and maintain study validity. The inclusion criteria were permanent anterior teeth (incisors and canines) with a documented history of dental trauma (concussion, subluxation, luxation, avulsion, or crown/root fractures) according to the International Association of Dental Traumatology (IADT) [10]; patients who underwent fixed orthodontic treatment after the trauma event; patients who completed at least 12 months of follow-up after orthodontic initiation; and patients aged typically between 8 and 18 years at the time of trauma. Exclusion criteria included patients who had received endodontic treatment before orthodontic treatment, patients with systemic conditions known to affect pulpal health (such as diabetes), trauma limited to enamel only, and cases with an orthodontic treatment duration of less than six months or follow-up shorter than 12 months.

The sample size was calculated using G*Power software (version 3.1; Heinrich-Heine-Universität Düsseldorf, Germany). Based on an expected pulp vitality loss of 20% in traumatized teeth and 5% in control teeth from a reference study, with a significance level of 0.05 and a power of 80%, and using the McNemar test for paired proportions appropriate for the split-mouth design, the minimum required sample size was estimated to be 54 teeth per group [11]. To compensate for possible attrition, 60 teeth with a history of trauma were included in each group. A total of 60 patients were selected for the contralateral paired group comparison.

Patients and teeth were grouped as follows: the primary group (trauma + orthodontics) included traumatized teeth subjected to OTM, and the control group consisted of non-traumatized teeth in the same patients (preferred for controlling individual factors). This grouping allowed a direct comparison of pulp vitality outcomes between exposed and non-exposed teeth. Data collection was performed by trained reviewers using a standardized proforma to extract relevant variables from the patient records. These included demographic details (age at trauma, age at orthodontic start, and sex), trauma characteristics (type and severity per IADT, time interval from trauma to orthodontic initiation, and apex maturity: open vs. closed), orthodontic parameters (direction of movement such as intrusion/extrusion/body, approximate force level, and treatment duration), and pulp vitality assessments (baseline and follow-up results from cold testing and electric pulp testing, along with any supplementary clinical notes on symptoms or need for endodontic intervention). All the data were anonymized and recorded on a secure electronic spreadsheet.

Pulp vitality was assessed primarily through sensibility testing, which is the standard clinical method used in endodontics. Cold testing involves the application of a refrigerant spray for 5-10 s on an isolated tooth surface, with responses categorized as positive (sharp/transient), negative (no response), or lingering. Electric pulp testing uses a calibrated device to record the threshold values or binary responses. A tooth was deemed non-vital if it showed consistent negative responses to both tests on repeated assessments, especially when supported by clinical symptoms or the initiation of root canal treatment. Positive responses without adverse changes indicate preserved vitality. Pulp sensibility testing was performed by a single calibrated examiner who was aware of group allocation; however, standardized testing protocols were followed to minimize measurement bias, and inter-examiner reliability was not applicable.

Statistical analyses were performed using statistical software (IBM SPSS, version 26.0; IBM Corp., Armonk, NY). The normality of continuous variables (age and time since trauma) was assessed using the Shapiro-Wilk test, which confirmed a normal distribution (p > 0.05), permitting the use of parametric tests where applicable. For the longitudinal comparison of pulp vitality responses within each group across different time intervals, the McNemar test was applied to account for the paired nature of the measurements. Between-group comparisons (traumatized vs. control) at each time point were performed using the chi-square test. Survival analysis of pulp vitality over the 12-month study period was conducted using the Kaplan-Meier method, and comparisons between groups were assessed using the log-rank test. Statistical significance was set at p < 0.05 for all analyses.

## Results

Sixty patients were enrolled, each contributing one previously traumatized permanent anterior tooth (traumatized group) and one non-traumatized contralateral anterior tooth (control group) to the fixed orthodontic treatment. The baseline demographic and trauma-related characteristics of the traumatized group, including age at orthodontic initiation, time since injury, and distribution of trauma types, are presented in Table 1. At baseline, pulp sensibility was positive in nearly all traumatized teeth and all control teeth, with no significant intergroup differences.

**Table 1:** Baseline characteristics of the study population (traumatized teeth group) Data are presented as mean ± standard deviation or number (percentage).

Parameters	Traumatized teeth group
Number of teeth (n)	60
Type of trauma (n, %)	
Enamel-dentin fracture	30 (50%)
Enamel-dentin-pulp fracture	12 (20%)
Subluxation	18 (30%)
Mean time elapsed since injury	3.8 ± 2.1 years
Mean age of patients (years)	16.5 ± 3.2
Baseline pulpal response (positive)	58 (96.7%)

Pulp vitality responses over the 12-month follow-up period showed a progressive decline in the traumatized group, whereas control teeth exhibited minimal change (Table 2). Significant between-group differences emerged starting at three months and persisted for 6 and 12 months (all p < 0.05). McNemar’s test confirmed significant longitudinal reductions in vitality within the traumatized group at baseline, with no comparable changes in the controls.

**Table 2:** Pulp vitality responses during orthodontic treatment at different time intervals *p < 0.05 denotes statistical significance using between-group comparisons by McNemar’s test for paired data.

Time interval	Traumatized group (n = 60), vital teeth (n, %)	Control group (n = 60), vital teeth (n, %)	Test statistic	p-value
Baseline (T0)	58 (96.7%)	60 (100%)	χ² = 2.03	0.154
3 months (T1)	54 (90.0%)	59 (98.3%)	χ² = 3.93	0.047*
6 months (T2)	50 (83.3%)	58 (96.7%)	χ² = 5.93	0.015*
12 months (T3)	48 (80.0%)	57 (95.0%)	χ² = 6.17	0.013*

**Figure 1:**
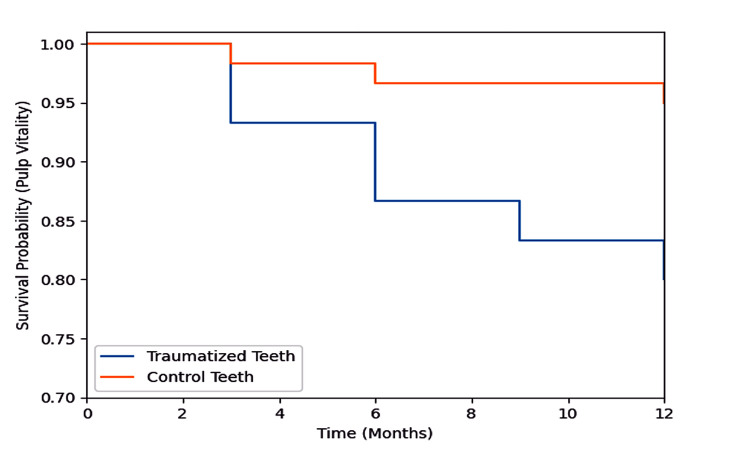
Kaplan-Meier survival curve of pulp vitality over 12 months

Kaplan-Meier survival analysis revealed shorter pulp vitality survival in the traumatized group than in the control group, with a statistically significant difference in the log-rank test (Table 3 and Figure 1). More vitality loss events occurred in the traumatized group during the study period.

**Table 3:** Kaplan-Meier survival analysis of pulp vitality over 12 months Events were defined as the first consistent loss of pulp sensibility leading to non-vital status. Survival time was censored at 12 months or at the end of follow-up.

Group	Total teeth (n)	Events (loss of vitality)	Censored (vital at 12 months)	Mean survival time (months)	95% confidence interval
Traumatized group	60	12	48	10.8	10.1–11.5
Control group	60	3	57	11.8	11.4–12.0
Overall	120	15	105	11.3	10.9–11.7
Log-rank test: χ² = 5.86, p = 0.016					

Adverse pulpal and periapical outcomes were more frequent in the trauma group (Table 4). Pulp necrosis was associated with a markedly elevated relative risk in traumatized teeth. Calcific metamorphosis occurred only in the traumatized group, although the difference was not statistically significant. All teeth exhibiting calcific metamorphosis remained vital during the follow-up period and did not progress to pulp necrosis. External root resorption rates were comparable between the groups. Overall, adverse events demonstrated a significantly higher relative risk in previously traumatized teeth that underwent orthodontic movement. These results demonstrate an increased susceptibility to compromised pulp vitality and related complications in traumatized teeth subjected to orthodontic forces relative to non-traumatized controls. However, the confidence interval for the relative risk was wide, reflecting the small number of events; therefore, this estimate should be interpreted with caution.

**Table 4:** Incidence of pulpal and periapical complications *Statistically significant (p < 0.05). Relative risks were calculated using standard methods for binary outcomes. p-values were obtained from Fisher’s exact test or χ², as appropriate. Total adverse events include any of the listed complications (some teeth had more than one event).

Complication/Outcome	Traumatized group (n = 60)	Control group (n = 60)	Relative risk (RR)	95% confidence interval	p-value
Pulp necrosis	8 (13.3%)	1 (1.7%)	8	1.03–62.1	0.035*
Pulp obliteration (calcific metamorphosis)	4 (6.7%)	0 (0.0%)	N/A	N/A	0.126
External root resorption	6 (10.0%)	5 (8.3%)	1.2	0.39–3.72	0.752
Total adverse events	18 (30.0%)	6 (10.0%)	3	1.28–7.05	0.009*

## Discussion

The findings of this prospective cohort study indicate that previously traumatized permanent anterior teeth are at a substantially greater risk of compromised pulp vitality and associated complications when subjected to OTM than non-traumatized contralateral teeth in the same patients. Pulp vitality declined progressively in the traumatized group during the follow-up period, whereas the control teeth remained largely stable. Survival analysis confirmed a significantly shorter duration of vitality maintenance in traumatized teeth, and the incidence of adverse pulpal and periapical events was markedly higher in the exposed group.

The biological basis for these observations lies in the pre-existing compromise of the pulpal neurovascular supply caused by initial dental trauma. Injuries involving the periodontal ligament disrupt apical blood flow and collateral circulation, often resulting in partial or complete pulp obliteration, reduced vascular reserve, and subclinical inflammatory changes [12]. Orthodontic forces further compress the periodontal ligament and induce transient ischemia within the pulp-dentin complex. In non-traumatized teeth, these effects are typically reversible under controlled physiological forces, owing to robust reparative mechanisms and adequate blood supply [3,7]. However, in previously traumatized teeth, the limited vascular capacity cannot adequately compensate for the additional mechanical and inflammatory stress, leading to irreversible pulpitis, necrosis, or delayed vitality loss. Contributing factors include the nature and severity of the original injury, maturity of the root apex, time elapsed since trauma, and cumulative effects of orthodontic remodeling [8,13]. Calcific metamorphosis, observed only in the traumatized group, represents an ongoing reparative process that may further restrict nutrient diffusion within the pulp chamber. In contrast, external root resorption rates were comparable between the groups, suggesting that prior trauma predominantly affects pulpal rather than periodontal tissues under the conditions studied.

These results are in agreement with the existing literature on the orthodontic-endodontic interface in traumatized teeth. Earlier studies focusing on specific movement types, such as intrusion or extrusion, have consistently reported elevated rates of pulp necrosis and vitality loss in teeth with a history of periodontal tissue involvement compared to non-traumatized or non-orthodontically treated traumatized teeth [11,13]. Previous reviews have similarly concluded that orthodontic movement increases the risk of pulpal complications in previously traumatized teeth, particularly when the initial injury affects the supporting structures or results in total pulp obliteration [14,15]. In contrast, Weissheimer et al. [16] conducted a systematic review to evaluate whether orthodontic tooth movement can induce pulp necrosis in vital teeth, focusing on studies that used reliable objective methods such as laser Doppler flowmetry, to assess pulpal blood flow. The authors concluded that orthodontic movements do not cause loss of pulp vitality or necrosis, with changes in pulpal blood flow being transient and reversible; however, this finding is supported by evidence of low to very low certainty due to methodological limitations and the risk of bias in the included studies.

Narrative reviews have further emphasized the predisposition of traumatized teeth to pulpal necrosis, canal calcification, and related sequelae during orthodontic treatment, although the strength of evidence remains moderate due to variability in study design, force application, and follow-up durations [7-9]. Lima et al. [17] conducted a systematic review to assess the efficacy of true vitality tests, such as pulse oximetry, laser Doppler flowmetry, and ultrasound Doppler flowmetry, in diagnosing the pulpal status of traumatized teeth compared to conventional sensibility tests, such as cold and electric pulp testing. The authors found limited high-quality evidence due to the high risk of bias and methodological deficiencies in the included studies, concluding that further well-designed research is needed to confirm the diagnostic accuracy and potential superiority of these objective vitality tests for traumatized teeth.

The strengths of the present investigation include the prospective cohort design with paired split-mouth controls, which effectively minimized confounding from individual patient variability, standardized pulp sensibility testing protocols, and a structured follow-up period that enabled survival analysis. The inclusion of a range of trauma types and a reasonable post-trauma interval enhances the applicability of the findings to typical clinical scenarios involving adolescents and young adults. The strengths of this study also include an adequate sample size determined by a priori power calculation and adherence to STROBE (Strengthening the Reporting of Observational studies in Epidemiology) guidelines, which enhance the methodological rigor and transparency of reporting.

These observations underscore the importance of interdisciplinary collaborations between orthodontists and endodontists. Clinicians should obtain a comprehensive trauma history before initiating treatment, consider deferring orthodontics for an adequate healing period when possible, apply light and controlled forces (particularly avoiding heavy intrusive mechanics on compromised teeth), and implement regular pulp vitality monitoring using multiple sensibility tests supplemented with periapical radiographs. Early recognition of adverse pulpal responses should prompt timely endodontic interventions to prevent further complications. Clinicians should obtain informed consent from patients, clearly explaining the elevated risk of pulp complications before initiating orthodontic treatment in previously traumatized teeth. The limitations of the study include its single-center design, absence of detailed quantification of orthodontic force levels, and lack of subgroup analyses stratified by trauma severity, apex maturity, or direction of tooth movement (such as intrusion, extrusion, and bodily movement), which may differentially influence pulpal vascular response. Additionally, reliance on clinical sensibility testing rather than more direct measures of pulpal blood flow represents a methodological constraint common to many similar investigations. Multicenter studies with standardized force protocols, longer observation periods, and objective vascular assessment tools are needed to further refine risk prediction and guide evidence-based management strategies.

## Conclusions

This prospective cohort study provided evidence that orthodontic tooth movement significantly increased the risk of pulp vitality loss and related complications in previously traumatized permanent anterior teeth compared with non-traumatized contralateral controls. The progressive decline in vitality and elevated incidence of adverse pulpal events highlighted the vulnerability of these teeth at the orthodontic-endodontic interface. These findings reinforce the need for cautious interdisciplinary management, including thorough trauma history assessment, light-force application, regular vitality monitoring, and patient education on heightened risks. Future multicenter research with standardized protocols and objective pulpal blood flow assessment is essential to refine the clinical guidelines and optimize outcomes for this high-risk population.

## References

[1] Wang Y, Chang H, Chen C, Yang X, Quan M, Liao X, Liao Z (2025). Traumatic dental injuries in children and adolescents presenting to a tertiary children's hospital in Shenzhen, China. BMC Oral Health.

[2] Lin S, Pilosof N, Karawani M, Wigler R, Kaufman AY, Teich ST (2016). Occurrence and timing of complications following traumatic dental injuries: a retrospective study in a dental trauma department. J Clin Exp Dent.

[3] Parashos P (2024). The orthodontic-endodontic interface: trauma and pulpal considerations. Br Dent J.

[4] Jeon HH, Teixeira H, Tsai A (2021). Mechanistic insight into orthodontic tooth movement based on animal studies: a critical review. J Clin Med.

[5] Golež A, Ovsenik M, Cankar K (2023). The effect of orthodontic tooth movement on the sensitivity of dental pulp: a systematic review and meta-analysis. Heliyon.

[6] Lam R, Goonewardene MS, Naoum S (2020). Pulp blood flow and sensibility in patients with a history of dental trauma undergoing maxillary expansion. Angle Orthod.

[7] Kindelan SA, Day PF, Kindelan JD, Spencer JR, Duggal MS (2008). Dental trauma: an overview of its influence on the management of orthodontic treatment. Part 1. J Orthod.

[8] Beck VJ, Stacknik S, Chandler NP, Farella M (2013). Orthodontic tooth movement of traumatised or root-canal-treated teeth: a clinical review. N Z Dent J.

[9] Bakkari A, Bin Salamah F (2022). Updated guidelines for the orthodontic management of traumatized and endodontically treated teeth: a review study. Cureus.

[10] Bourguignon C, Cohenca N, Lauridsen E (2020). International Association of Dental Traumatology guidelines for the management of traumatic dental injuries: 1. fractures and luxations. Dent Traumatol.

[11] Bauss O, Röhling J, Sadat-Khonsari R, Kiliaridis S (2008). Influence of orthodontic intrusion on pulpal vitality of previously traumatized maxillary permanent incisors. Am J Orthod Dentofacial Orthop.

[12] Kvinnsland I, Heyeraas KJ, Byers MR (1992). Effects of dental trauma on pulpal and periodontal nerve morphology. Proc Finn Dent Soc.

[13] Andreasen JO, Bakland LK, Andreasen FM (2006). Traumatic intrusion of permanent teeth. Part 2. A clinical study of the effect of preinjury and injury factors, such as sex, age, stage of root development, tooth location, and extent of injury including number of intruded teeth on 140 intruded permanent teeth. Dent Traumatol.

[14] Javed F, Al-Kheraif AA, Romanos EB, Romanos GE (2015). Influence of orthodontic forces on human dental pulp: a systematic review. Arch Oral Biol.

[15] Duarte PH, Weissheimer T, Michel CH, Só GB, da Rosa RA, Só MV (2023). Do orthodontic movements of traumatized teeth induce dental pulp necrosis? A systematic review. Clin Oral Investig.

[16] Weissheimer T, Silva EJ, Pinto KP, Só GB, Rosa RA, Só MV (2021). Do orthodontic tooth movements induce pulp necrosis? A systematic review. Int Endod J.

[17] Lima TF, Dos Santos SL, da Silva Fidalgo TK, Silva EJ (2019). Vitality tests for pulp diagnosis of traumatized teeth: a systematic review. J Endod.

